# Factors Affecting Robotic Partial Nephrectomy Conversion to Radical Nephrectomy: A Retrospective Multi-Institutional Analysis in the Michigan Urologic Surgery Improvement Collaborative (MUSIC)

**DOI:** 10.7759/cureus.20477

**Published:** 2021-12-17

**Authors:** Benjamin Goldman, Michael Rudoff, Ji Qi, David Wenzler

**Affiliations:** 1 Urology, Ascension Providence Hospital, Southfield, USA; 2 Urology, University of Michigan, Ann Arbor, USA

**Keywords:** robotic partial nephrectomy, michigan urologic surgery improvement collaborative, risk factors, radical nephrectomy, conversion to open

## Abstract

Objective: To identify factors related to the conversion of robot-assisted partial nephrectomy (RPN) to robot-assisted radical nephrectomy (RRN) based on data collected by a statewide database in Michigan.

Methods: Using the Michigan Urological Surgery Improvement Collaborative-Kidney mass: Identifying and Defining Necessary Evaluation and therapY (MUSIC-KIDNEY) database we identified 574 patients for whom RPN was planned. Patient and tumor characteristics were obtained including body mass index (BMI), Charlson comorbidity index (CCI), RENAL nephrometry score, tumor size, and pathologic staging. Treating centers were subdivided by annualized case volume and academic status. Bivariate and multivariate analyses were performed to assess the impact of these factors on the risk of conversion to RRN from RPN.

Results: The conversion rate of RPN to RN was 5.75% (33/574). The difference in RENAL nephrometry score, tumor stage, and size reached statistical significance on bivariate analysis (p<0.001). The tumor stage also reached statistical significance on multivariate analysis [odds ratio (OR); 95%CI (8.97; 3.93-20.48) p<0.001]. The conversion rate was lower among high-volume versus low-volume practices; however, statistical significance was not reached [5.2% (27/520) vs.11% (6/54); p=0.11].

Conclusions: Patient factors such as tumor size and renal nephrometry score are likely related to the conversion of RPN to RRN decisions. The data shows that Michigan urologists appear to appropriately assess intra-operative findings and convert to RRN in cases of more advanced kidney tumors. Lower volume centers appear to trend towards a higher conversion rate. Continued quality improvement tracking analysis may further clarify this relationship.

## Introduction

Contemporary literature supports partial nephrectomy as a preferred approach to treating low stage, localized kidney cancer [1,2]. Nephron sparing surgery (NSS) has been shown to have excellent oncologic control in appropriately selected patients while preserving renal function in comparison to radical nephrectomy [3-5]. There is also evidence to support NSS for larger, and even higher stage tumors [6,7]. As robot-assisted surgery has become widespread in urologic practice, there is strong evidence showing acceptable oncologic outcomes with robot-assisted partial nephrectomy as well as improved perioperative outcomes for these cases. Robot-assisted partial nephrectomy (RPN) is recommended by current guidelines for renal tumors amenable to partial nephrectomy [8].

Any partial nephrectomy may be converted to a radical nephrectomy intra-operatively if a surgeon is concerned about safety or oncologic risk. This is also true regarding robot-assisted partial nephrectomy. Recent literature suggests that the rate of conversion from RPN to robot-assisted radical nephrectomy (RRN) ranges from 0.7-5% [9-13]. Most published studies reporting these rates include data from high volume centers. In this study we sought to identify factors that affect conversion from RPN to RRN using a prospectively maintained database compiled by institutions of varying volume.

## Materials and methods

The Michigan Urological Surgery Improvement Collaborative-Kidney mass: Identifying and Defining Necessary Evaluation and therapY (MUSIC-KIDNEY) conception and data collection methods have been previously outlined by the collaborative in 2019 [14]. The MUSIC coordinating center is responsible for overall administration and management of collaborative activities. One urologist per practice serves as the clinical champion with responsibilities that include oversight of local data collection and leadership around the local implementation of quality improvement activities. Data abstractors recorded 122 data points at a single time point (≥120 days after initial consultation).

For the purposes of this study, we included all patients diagnosed with clinical stage cT1a or cT1b renal mass (measuring less than 4 cm or 4-7cm respectively) and for whom RPN was planned captured by the MUSIC database [15]. The primary endpoint was the conversion from RPN to RRN at the time of surgery.

For each patient, the following variables were extracted and included for analysis: age, body mass index (BMI), Charlson comorbidity index (CCI), tumor size (clinical staging), tumor complexity using RENAL nephrometry score [16], and preoperative creatinine value. Two practice-level characteristics were also included in the analysis. The first characteristic is practice type, which is classified as academic vs private/community-based vs hybrid. The second is annualized surgical volume on partial nephrectomy, and each practice was dichotomized into low (<24/year) vs high volume (>24/year) as previously defined by Leow et al. [17]. For patients undergoing either partial or radical nephrectomy, the following pathological staging outcomes were available: the presence of fat invasion (pT3a), Gerota’s fascia invasion (pT4), vascular invasion (pT3b), adrenal invasion (pT4 or M1).

Patient-level and practice-level characteristics were compared between patients converted from RPN to RRN and those receiving successful RPN, using Chi-squared test for categorical variables and Wilcoxon rank-sum test for continuous measures. Practice-level variation in the proportion of patients converting to RRN was examined. Pathological outcomes between patients undergoing RRN vs RPN were also compared through the Chi-squared test or Fisher’s exact test. A mixed-effects logistic regression model was performed to identify factors associated with converting to RRN. The model included predictors such as patient age, BMI, comorbidity, and tumor size/staging, and random intercepts for each surgeon to account for within-surgeon correlation. All the analyses were performed using SAS 9.4 (SAS Institute, Cary, USA), and statistical significance was set at p=0.05.

## Results

Between July 2016 and Jan 2021, a total of 574 patients were diagnosed with T1 renal masses and scheduled for an RPN in the MUSIC registry. The rate of conversion from RPN to RRN was 5.75% (33/574). No open conversions were documented. Table 1 compares the patient and tumor characteristics of this cohort by conversion status. Patients with a larger tumor (T1b vs T1a) or that of higher complexity were more likely to convert to an RRN (p<0.01 for each). Preoperative creatinine was slightly higher in the group that was converted to RRN (median 1.0 vs 0.9, p=0.03). 

**Table 1:** Clinical and demographic characteristics by conversion status RRN = robot-assisted radical nephrectomy; BMI = Body Mass Index; IQR = interquartile range; T1a and T1b = Clinical stages of renal masses

Variables	Not converted to RRN	Converted to RRN	p-Value
No. patients	541	33	
Charlson comorbidity index			
0	333 (94.9%)	18 (5.1%)	0.683
1	106 (93.8%)	7 (6.2%)	
>=2	102 (92.7%)	8 (7.3%)	
RENAL nephrometry score			
Low	161 (98.2%)	3 (1.8%)	<0.001
Intermediate	162 (95.3%)	8 (4.7%)	
High	15 (55.6%)	12 (44.4%)	
Tumor size			
T1a	427 (97.5%)	11 (2.5%)	<0.001
T1b	114 (83.8%)	22 (16.2%)	
Practice type			
Academic	106 (97.2%)	3 (2.8%)	0.261
Private/Community-based	27 (96.4%)	1 (3.6%)	
Hybrid	408 (93.4%)	29 (6.6%)	
Age, median (IQR)	59.0 (49.0-67.0)	63.0 (56.0-69.0)	0.079
BMI, median (IQR)	30.3 (27.0-35.6)	31.9 (28.7-36.3)	0.410
Tumor size, median (IQR)	2.8 (2.0-3.7)	4.7 (3.5-5.6)	<0.001
Preop creatinine, median (IQR)	0.9 (0.8-1.1)	1.0 (0.9-1.2)	0.030

**Figure 1:**
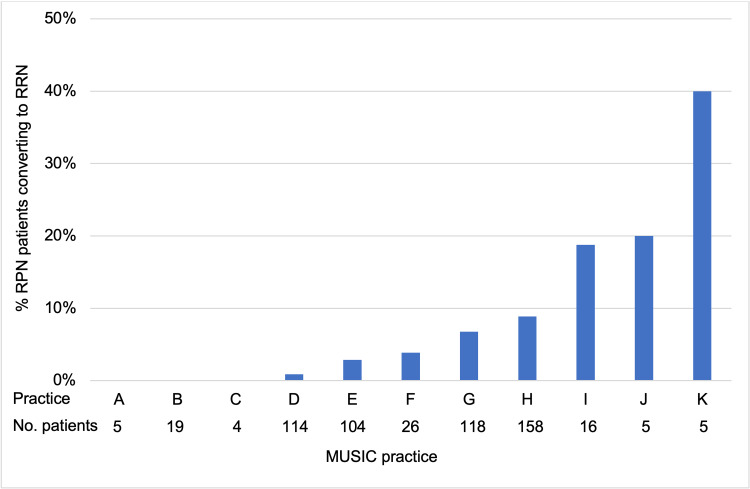
Practice-level rate of conversion to RRN from RPN RPN = Robotic partial nephrectomy; RRN = Robotic radical nephrectomy; MUSIC = Michigan Urological Surgery Improvement Collaborative

A total of 11 practices contributed data to the study sample. The conversion rate among these practices ranges from 0% to 40% (Figure 1). Of these, six were low-volume practices and five were high-volume. The high- and low-volume practices comprised 90.5% (520/574) and 9.5% (54/574) of the cases, respectively. Although not statistically significant, the conversion rate was lower among high-volume compared to low-volume practices (27/520=5.2% vs. 6/54=11%; p=0.11). The majority of cases were performed at hybrid centers (437/574) of which 6.6% (29/437) were converted from RPN to RRN. Conversion rates at academic and community centers were lower (2.8% and 3.6%, respectively), but the difference did not reach statistical significance (p=0.261).

On multivariable analysis (Table 2), patients with a T1b tumor were more likely to convert to an RRN (OR=8.97, p<0.01). No other variables were identified to be significantly associated with conversion on multivariable analysis. 

**Table 2:** Multivariable analysis of factors associated with converting to RPN to RRN CCI = Charlson Comorbidity Index; RPN = Robotic partial nephrectomy; RRN = Robotic radical nephrectomy; T1a and T1b = Clinical stages of renal masses; BMI = Body Mass Index

Variable	OR	95% CI
CCI 1 vs 0	1.08	(0.40, 2.92)
CCI >=2 vs 0	1.35	(0.50, 3.67)
T1b vs T1a tumor	8.97	(3.93, 20.48)
Age	1.03	(0.99, 1.07)
BMI	0.99	(0.93, 1.05)

A significant difference in pathological outcomes was observed between converted vs RPN cases. Among the 33 converted cases, the fat invasion was present in 24.2% (8/33) of RRN specimens, which was significantly more than those found on RPN specimens (6.3% p<0.001). The rate of pT4 disease was 6% vs 1.5% for converted vs RPN cases, respectively (p=0.11) A significantly higher rate of vascular invasion was observed among converted cases compared to RPN patients (21.2% vs.5.2%p=0.002). The direct adrenal invasion was found in 3% (1/33) of the converted cases whereas no RPN cases had adrenal invasion (p=0.06).

## Discussion

The MUSIC-KIDNEY collaborative database provides a system for statewide tracking and analysis of renal surgery performed in Michigan. This has offered the opportunity to evaluate perioperative outcomes of high and low-volume institutions. The findings reported here are similar to previous studies with regard to the overall RPN to RRN conversion rate. There is not a clear consensus on which perioperative factors appear to impact this rate the most. Petros et al. and Kara et al. showed tumor size, stage, and complexity to be significantly associated with conversion [10,11]. Arora et al. reported a significant association with patient-related factors, such as CCI and BMI [13]. Petros et al. reported case-specific reasons for conversion such as an invasive tumor or blood loss [11]. In our study, more than half of the final pathology data indicates invasive tumors were present in the cases that were converted to RRN which reaches statistical significance compared to the RPN group. Therefore, although specific surgeon decision documentation is lacking, we may be able to infer that in many of these cases the decision to convert to RRN was related to uncovering intraoperative evidence to suggest higher stage tumors in those cases. Renal masses can be assessed with the RENAL nephrometry score and have been validated as a risk-assessment tool regarding performing partial nephrectomy versus radical nephrectomy [17]. Our data further support this assessment since tumors with high RENAL nephrometry scores were statistically more likely to have been converted. 

Although the clinical importance of the significant difference in creatinine reported here is questionable, preoperative assessment of renal function is important when considering treatment options and counseling patients found to have renal masses. As the prevalence of diabetes mellitus and hypertension and associated kidney disease continue to rise in the United States, pressure to preserve nephrons in these patients leads clinicians to perform NSS whenever possible since radical nephrectomy has been shown to independently predict new onset and progression of kidney disease [3,18]. Also, a recent innovation in minimally invasive surgery has placed greater emphasis on performing PN in a minimally invasive fashion due to an improved length of stay, blood loss, and transfusion rates as well as renal and oncologic outcomes [9]. 

Clinical registries have an important role in urologic research and quality improvement initiatives by providing data on granular outcome measures and patient-reported outcomes that may be lacking in claims-based data [19]. Although national databases exist, long-term follow up is lacking in some, such as the American College of Surgeons National Surgical Quality Improvement Program (ACS NSQIP), and the variables automatically abstracted from the electronic medical record are still somewhat limited in others, such as American Urological Association Quality (AQUA) Registry. The MUSIC database was designed for regional collaboration to identify variations in practice that may be targeted to ultimately raise the level of care provided to patients with urologic pathology in Michigan. Data are collected prospectively and follow-up is indefinite. Recent findings for outcome variation from not only renal cancer [20], but also prostate cancer diagnosis, surveillance, and treatment [21-23] identify specific targets to be addressed and can result in less outcome variation [24].

Most of the contemporary urologic literature in the United States is based on data from high volume and/or major academic centers, especially regarding operative outcomes. Although centralization of care to these centers can be more easily tracked, representation of outcomes from lower volume and/or community-based centers is lacking. There is limited data available comparing outcome differences between these types of centers. A literature review of over four thousand patients showed excellent outcome measures of RPN with no meaningful differences between high and low volume centers [16]. In our subgroup analysis, the low-volume centers did have a higher rate of conversion compared to high-volume centers, but statistical significance was not reached (data not shown). Most cases were performed at hybrid centers and although conversion rates were lower at the community and academic centers, this is more likely due to differences in volume and a statistically significant difference was not reached.

This study is limited by its retrospective nature and individual records may be inconsistent in the granularity of detail available for abstraction. Although we were able to demonstrate a statistically significant difference in RENAL nephrometry score between groups, some records were incomplete regarding this datapoint. The records revealed some information about perioperative factors that may have played a role in the decision to convert; however, explicit documentation from the surgeon was not consistently available. Therefore, our data is limited regarding some outcome measures of interest. Although the total cohort size reported here is well over 500 cases, subgroup analysis is limited by the relatively low total number of converted cases. The data may not be powered to show statistically significant differences for some subgroup analyses. It appears to trend towards a higher conversion rate for low-volume centers. The MUSIC database will continue to compile the outcomes of these cases and future analysis may elucidate this difference.

## Conclusions

Patients with small renal masses considering minimally invasive surgical management with RPN should be counseled on the low risk of conversion to RRN. The data reported here suggest the risk of conversion is significantly higher in patients with larger, complicated tumors. There appears to be an increased rate of conversion to RRN at lower volume centers. Continued data collection and investigation are needed to clarify this relationship. 
